# Alpha-1 Antitrypsin-Induced Endoplasmic Reticulum Stress Promotes Invasion by Extravillous Trophoblasts

**DOI:** 10.3390/ijms22073683

**Published:** 2021-04-01

**Authors:** Kanoko Yoshida, Kazuya Kusama, Yuta Fukushima, Takako Ohmaru-Nakanishi, Kiyoko Kato, Kazuhiro Tamura

**Affiliations:** 1 Department of Endocrine Pharmacology, Tokyo University of Pharmacy and Life Sciences, Tokyo 192-0392, Japan; y164241@toyaku.ac.jp (K.Y.); 3110furk@gmail.com (Y.F.); hiro@toyaku.ac.jp (K.T.); 2 Department of Obstetrics and Gynecology, Graduate School of Medical Sciences, Kyushu University, Fukuoka 812-8582, Japan; ohmaru2012@gmail.com (T.O.-N.); kkato@med.kyushu-u.ac.jp (K.K.)

**Keywords:** extravillous trophoblast, alpha-1 antitrypsin, high-temperature requirement A serine peptidase 1, endoplasmic reticulum stress, cell invasion

## Abstract

Alpha-1 antitrypsin (A1AT) is a glycoprotein that has been shown to protect tissues from proteolytic damage under various inflammatory conditions. Several studies show that A1AT may be associated with pre-eclampsia. However, the role of A1AT expression in placental physiology is not fully understood. In the present study, we aim to characterize the expression and function of placental A1AT. A1AT knockdown is found to reduce the expression of the serine protease HTRA1 in a trophoblast cell line. In addition, A1AT overexpression (A1AT-OE) increases the expression of HTRA1, IL6, CXCL8, and several markers of endoplasmic reticulum (ER) stress. Treatment with tunicamycin or thapsigargin, which induces ER stress, increases HTRA1 expression. Furthermore, immunohistochemistry reveals that HTRA1 is expressed in trophoblasts and the endometrial decidual cells of human placentas. An invasion assay shows that A1AT and HTRA1 stimulate cell invasion, but treatment with the ER stress inhibitors reduces the expression of HTRA1 and ER stress markers and prevents cell invasion in A1AT-OE trophoblasts. These results suggest that endogenous A1AT regulates inflammatory cytokine expression and HTRA1-induced trophoblast invasion via the induction of ER stress. It is concluded that an imbalance in the functional link between A1AT and ER stress at the maternal–fetal interface might cause abnormal placental development.

## 1. Introduction

The placenta plays a major role in feto-maternal communication and the maintenance of pregnancy. In early pregnancy, mononuclear cytotrophoblasts fuse to multinucleated syncytiotrophoblasts that cover the floating chorionic villi, or differentiate into extravillous trophoblasts, which are capable of invading the endometrium and develop from anchoring villi. Impairment of trophoblast cell invasion into the endometrium causes serious complications during pregnancy, including hypertensive disorder of pregnancy (HDP), which is mainly caused by insufficient invasion by extravillous trophoblast cells. However, the detailed molecular mechanisms of the development of HDP and pre-eclampsia (PE) remain to be determined. Nevertheless, inadequate invasion of extravillous trophoblasts into the myometrium of the uterus and inappropriate remodeling of the uterine spiral arterioles are thought to be involved in these disorders [1]. There are several mechanisms that may contribute to trophoblast dysfunction, including ischemic placenta, dysregulation of angiogenesis, and excessive oxidative stress [2].

Alpha-1 antitrypsin (A1AT), the archetypal member of the serpin superfamily, is encoded by the SERPINA1 gene. This serine protease inhibitor is present at high circulating concentrations in inflammatory diseases [3]. Previous studies have shown that a reduction of A1AT protein in endometriosis-like lesions exacerbates the inflammatory response in mice [4]. It has been shown that urinary A1AT may represent a marker of the severity of PE [5]. Furthermore, A1AT may reduce organ damage in a serine protease activity-independent fashion [6], although interaction with high-temperature requirement A serine peptidase 1 (HTRA1) may be involved [7]. Several HTRAs are expressed in trophoblasts [8,9], but the roles of A1AT and HTRAs in placental physiology and disorders are not fully understood.

The excessive deficiency and polymerization of A1AT are related to emphysema, which involves the intracellular accumulation of mutated protein, leading to endoplasmic reticulum (ER) stress [10]. The unfolded protein response (UPR) is a response to ER stress that is conserved among all mammalian species [11] and that leads to an increase in the production of molecular chaperones. Impairments in ER homeostasis can occur under physiological and/or pathological conditions, and the UPR can involve the activation of various signaling pathways, including pro-inflammatory pathways [12]. In our previous study, we showed that A1AT regulates the production of pro-inflammatory adipokines and other pro-inflammatory factors through UPR-related genes in human omental adipocytes [13]. Although ER stress may influence endometrial invasion by extravillous trophoblasts [14], it remains unclear whether A1AT and/or HTRA1 are involved in ER stress in trophoblasts. We hypothesize that intracellular A1AT may regulate inflammatory cytokine expression and HTRA1-induced invasion via the induction of ER stress in trophoblasts. Our hypothesis also implies that the roles of intracellular A1AT and circulating A1AT may be different. Abnormal regulation of A1AT expression could cause defective trophoblast invasion, leading to HDP and PE. To test this hypothesis, we determine the effects of the knockdown and overexpression of A1AT on the expression of inflammation- and ER stress-related factors in human extravillous trophoblast HTR8/SVneo cells and whether pharmacological stimulation or inhibition of UPR signaling affects the invasion of trophoblasts.

## 2. Results

### 2.1. Alpha-1 Antitrypsin (A1AT) Regulates the Expression of HTRA1 in an Extravillous Trophoblast Cell Line

**Figure 1:**
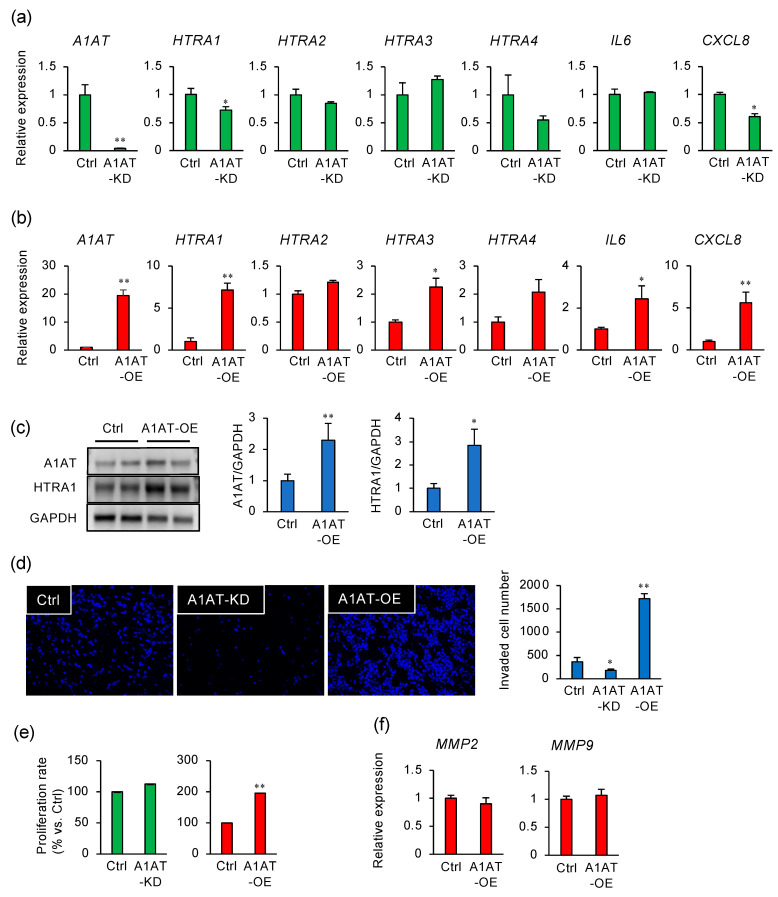
Alpha-1 antitrypsin (A1AT) regulates the expression of HTRA1 in an extravillous trophoblast cell line. (**a**) RNA was extracted from human extravillous trophoblast HTR8/SVneo cells that had been transfected with non-targeting control siRNA (Ctrl, 50 nM) or *A1AT* siRNA (A1AT knockdown (A1AT-KD), 50 nM) 24 h previously. The effect on *A1AT*, *HTRA*s, *IL6*, and *CXCL8* mRNA expression was determined by qPCR (*n* = 3 per treatment). *GAPDH* mRNA was used as the reference gene. The values are the means ± SEMs of three independent experiments. * *p* < 0.05, ** *p* < 0.01. (**b**) mRNA expression of *A1AT*, *HTRA*s, *IL6*, and *CXCL8* in HTR8/SVneo cells that had been transfected with an empty vector (Ctrl) or an A1AT expression vector (A1AT overexpression (A1AT-OE)). *GAPDH* mRNA was used as the reference gene. The values are the means ± SEMs of three independent experiments. * *p* < 0.05, ** *p* < 0.01. (**c**) Lysates prepared from HTR8/SVneo cells treated as above were subject to immunoblotting. GAPDH served as the loading control. The values are the means ± SEMs of three independent experiments. * *p* < 0.05, ** *p* < 0.01. (**d**) A1AT-KD and A1AT-OE HTR8/SVneo cells were incubated in Matrigel-coated transwells for 48 h. Representative images of the cells that passed through the transmembranes are shown in the panels on the left. The numbers of cells that passed through the transmembranes were counted and are presented as the means ± SEMs of the three independent experiments. * *p* < 0.05, ** *p* < 0.01. (**e**) A1AT-KD or A1AT-OE HTR8/SVneo cells were cultured for 48 h and the numbers of cells were determined using a WST-8 assay. ** *p* < 0.01. (**f**) The expression of *MMP2* and *MMP9* in A1AT-OE HTR8/SVneo cells was measured. *GAPDH* was used as the reference gene. The values are the means ± SEMs from three independent experiments.

In human placental tissue, A1AT may interact directly with HTRA1 [4,7] and suppress the inflammatory response [4,7]. The effect of A1AT knockdown (KD) or overexpression (OE) on the expression of *HTRAs* and the pro-inflammatory genes *IL6* and *CXCL8* was determined in HTR8/SVneo cells (Figure 1a,b). A1AT-KD reduced the expression of *HTRA1*, *CXCL8*, and *A1AT* (Figure 1a), whereas A1AT-OE increased the expression of *HTRA1*, *HTRA3*, *HTRA4, IL6*, and *CXCL8* in the trophoblast cells (Figure 1b). Furthermore, A1AT-OE increased the amount of the HTRA1 protein (Figure 1c). As HTRA1 regulates the invasion of trophoblast cells [15], we evaluated the effect of A1AT on that invasion. A1AT-KD reduced the number of invading cells, whereas A1AT-OE increased the number (Figure 1d). In addition, A1AT-OE trophoblasts exhibited an approximately two-fold higher rate of proliferation (Figure 1e), but A1AT-OE did not affect the expression of the trophoblast invasion markers *MMP2* and *MMP9* (Figure 1f).

### 2.2. HTRA1 Regulates Invasion by Extravillous Trophoblast Cells

**Figure 2:**
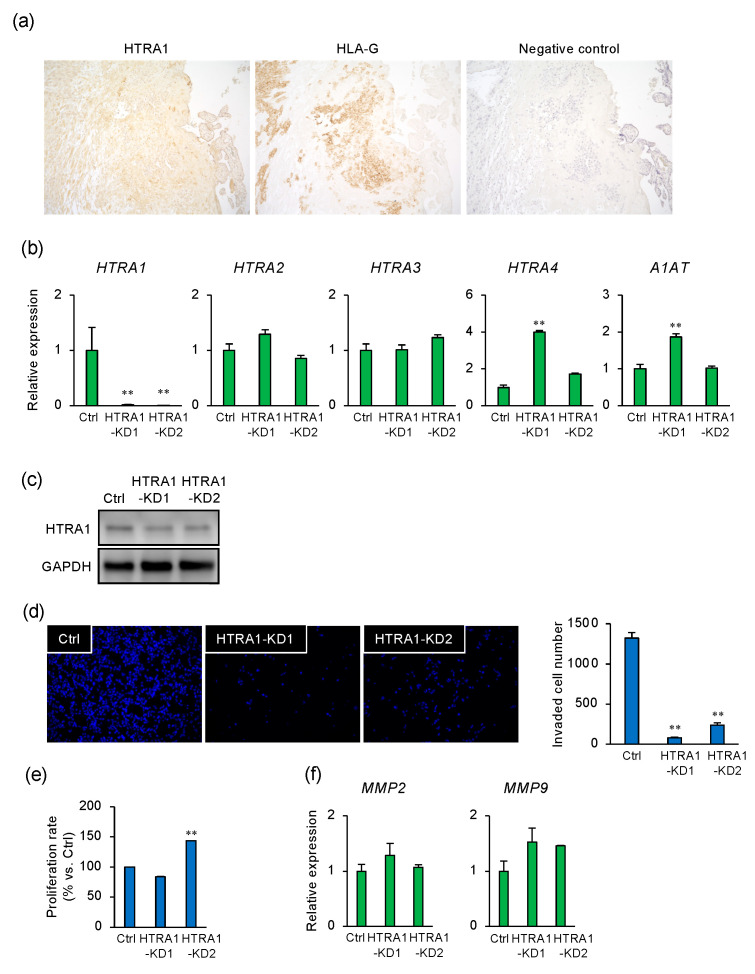
HTRA1 regulates invasion by extravillous trophoblast cells. (**a**) Tissue sections of human placenta were stained with anti-HTRA1, HLA-G (positive control for extravillous trophoblast), or normal rabbit IgG (negative control). (**b**,**c**) Extravillous trophoblast HTR8/SVneo cells were transfected with two different *HTRA1* siRNAs (HTRA1-KD1 and -KD2, each 50 nM) and incubated for 24 h. (**b**) The mRNA expression of *HTRA*s and *A1AT* was determined (*n* = 3). *GAPDH* was used as the reference gene. The values are the means ± SEMs. ** *p* < 0.01. (**c**) Lysates prepared from HTR8/SVneo cells that had been transfected with HTRA1-KD1 or -KD2 were subject to immunoblotting. GAPDH served as a loading control. (**d**) HTR8/SVneo cells that had been transfected with HTRA1-KD1 or -KD2 were incubated in Matrigel-coated transwells for 48 h. Representative images of the cells that passed through the transmembranes are shown in panels on the left. The numbers of cells that passed through the transmembranes were counted and are presented as the means ± SEMs of three independent experiments. ** *p* < 0.01. (**e**) HTR8/SVneo cells that had been transfected with HTRA1-KD1 or -KD2 were cultured for 48 h, and then the numbers of cells were determined using a WST-8 assay. ** *p* < 0.01. (**f**) The mRNA expression of *MMP2* and *MMP9* in HTR8/SVneo cells that had been transfected with HTRA1-KD1 or -KD2 was measured. *GAPDH* was used as the reference gene. The values are the means ± SEMs of the three independent experiments.

To characterize the expression of HTRA1 in the placenta, term placental tissue was immunostained for HTRA1 and HLA-G, a marker of the extravillous trophoblast. HTRA1 was localized in the extravillous trophoblast, syncytiotrophoblast, and endometrial decidual cells (Figure 2a). To ascertain whether HTRA1 regulates invasion by extravillous trophoblast cells, the effects of the knockdown of HTRA1 were determined using two siRNAs (HTRA1-KD1 and -KD2) that specifically recognize *HTRA1* mRNA sequences in different regions. The *HTRA1* siRNAs reduced HTRA1 mRNA and protein expression but did not change the expression of HTRA paralogs (Figure 2b,c). Both *HTRA1* siRNAs markedly inhibited cell invasion (Figure 2d). A slight increase in cell proliferation was observed in cells that had been transfected with HTRA1-KD2 (Figure 2e), but there was no effect on the expression of *MMP2* or *MMP9* (Figure 2f).

### 2.3. A1AT-Induced HTRA1 Expression and Cell Invasion Are Regulated by UPR Signaling

**Figure 3:**
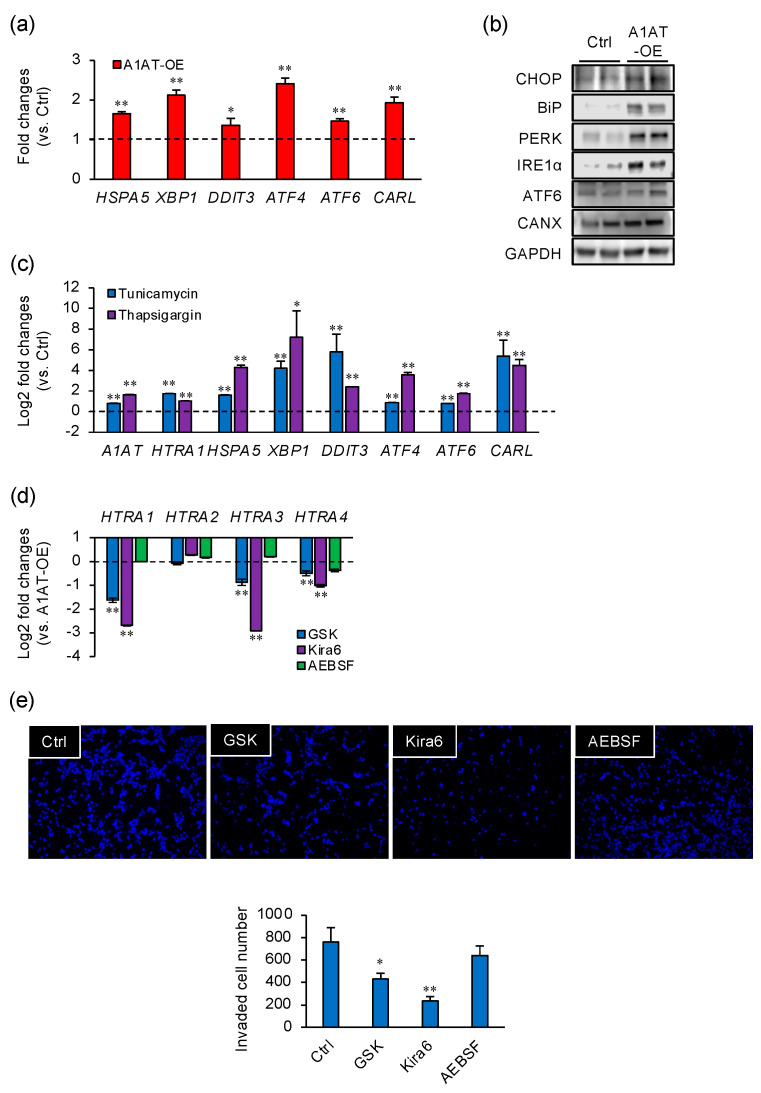
A1AT-induced HTRA1 expression and cell invasion are regulated by unfolded protein response (UPR) signaling. (**a**) The mRNA expression of endoplasmic reticulum (ER) stress markers in HTR8/SVneo cells that had been transfected with A1AT-OE was measured. *GAPDH* was used as the reference gene. The values are the means ± SEMs of three independent experiments. * *p* < 0.05, ** *p* < 0.01. (**b**) The protein levels of ER stress markers were measured by immunoblotting lysates from A1AT-OE HTR8/SVneo cells. GAPDH served as a loading control. (**c**) HTR8/SVneo cells were treated with tunicamycin (5 μM) or thapsigargin (100 nM) for 48 h, and then the mRNA expression of *A1AT*, *HTRA1*, and ER stress markers was measured using qPCR. *GAPDH* was used as the reference gene. The values are the means ± SEMs of three independent experiments. * *p* < 0.05, ** *p* < 0.01. (**d**) A1AT-OE HTR8/SVneo cells were treated with GSK2606414 (GSK, 250 nM), Kira6 (1 μM), or AEBSF (300 μM) for 24 h, and then the mRNA expression of *HTRA*s was measured. *GAPDH* was used as the reference gene. The values are the means ± SEMs of three independent experiments. ** *p* < 0.01. (**e**) A1AT-OE HTR8/SVneo cells were treated with GSK2606414 (GSK, 250 nM), Kira6 (1 μM), or AEBSF (300 μM) in Matrigel-coated transwells for 24 h. Representative images of the cells that passed through the transmembranes are shown in the panels on the left. The numbers of cells that passed through the transmembranes were counted and are presented as the means ± SEMs of three independent experiments. * *p* < 0.05, ** *p* < 0.01.

It has been reported that A1AT induces ER stress [10]. To investigate the effect of A1AT-OE on the induction of ER stress in trophoblast cells, the expression of ER stress markers was measured in A1AT-OE extravillous trophoblast cells. The expression of the ER stress marker genes HSPA5, XBP1, DDIT3, ATF4, ATF6, and CARL was increased by A1AT-OE (Figure 3a), as were the levels of ER stress marker proteins (Figure 3b). Next, we determined the effects of the ER stress inducers tunicamycin and thapsigargin on HTRA expression (Figure 3c). Treatment with either ER stress inducer increased the expression of HTRA1 and that of ER stress markers. Moreover, A1AT expression was increased by tunicamycin or thapsigargin treatment (Figure 3c). To further investigate the effect of ER stress on the expression of HTRA1, A1AT-OE extravillous trophoblasts were treated with GSK, Kira6, or AEBSF, the ER stress inhibitors of PERK, IRE1α, and ATF6, respectively. GSK and Kira6 reduced the capacity of A1AT-OE to induce HTRA1, HTRA3, and HTRA4 expression, whereas AEBSF had no effect (Figure 3d). In addition, GSK and Kira6 inhibited cell invasion (Figure 3e).

## 3. Discussion

**Figure 4:**
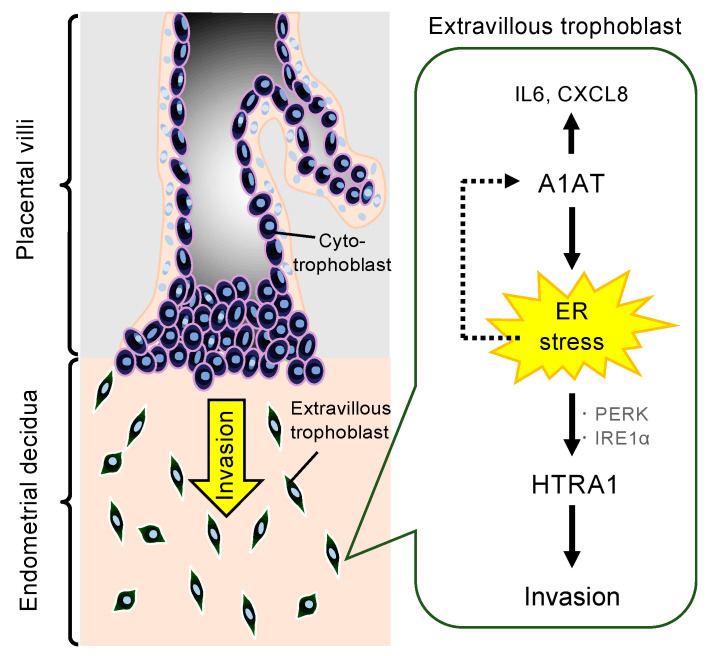
Diagram illustrating the proposed mechanism linking A1AT and invasion by extravillous trophoblast cells. Abnormal A1AT expression induces ER stress, which leads to the upregulation of HTRA1 and results in invasion by trophoblast cells. ER stress increases A1AT expression. In addition, A1AT stimulates *IL6* and *CXCL8* expression.

This study suggests that endogenous A1AT regulates local inflammation and HTRA1-induced cell invasion via the induction of ER stress in extravillous trophoblasts. We found that A1AT-KD reduced HTRA1 expression, whereas A1AT-OE increased its expression, but not that of HTRA paralogs, which implies that A1AT specifically regulates HTRA1 expression. In addition, A1AT-KD reduced invasion by trophoblast cells, whereas A1AT-OE increased invasion without stimulating cell proliferation or an increase in the expression of MMP2 or MMP9. Knocking down HTRA1 also significantly reduced trophoblast invasion. Notably, the expression of several ER stress markers was induced in A1AT-OE trophoblast cells. In addition, the pharmacological inducers of ER stress—tunicamycin and thapsigargin—increased the expression of HTRA1 and A1AT, along with that of ER stress markers. However, the A1AT-OE-induced expression of HTRA1 was largely prevented by treatment with the UPR inhibitors GSK or Kira6, but not AEBSF, and both GSK and Kira6 inhibited invasion. These results indicate that A1AT may transcriptionally regulate HTRA1 expression through the ER stress-induced UPR, and specifically via PERK and IRE1α, leading to invasion by extravillous trophoblast cells (Figure 4).

Alpha 2 macroglobulin, matrix Gla protein, and type 2 collagen have to date been identified as substrates of HTRA1 [16]. A1AT is a serine protease inhibitor that might also interact with HTRA1 [7]. We evaluated the interaction between A1AT and HTRA1 using an immunoprecipitation assay but could not demonstrate the binding of A1AT to HTRA1 (data not shown). Jonigk et al. [6] have demonstrated that A1AT reduces organ damage in a serine protease activity-independent fashion, but the intracellular accumulation of A1AT protein induces ER stress [10]. Consistent with this, in the present study, A1AT-OE increased ER stress marker expression. ER stress requires three major ER-spanning transmembrane proteins, PERK, IRE1α, and ATF6, all of which bind to BiP (encoded by *HSPA5*) [11]. The activation of PERK by the dissociation of BiP increases ATF4 expression, which directly upregulates CHOP (encoded by *DDIT3*), while the activation of IRE1α by the dissociation of BiP upregulates spliced XBP1 [17]. In the present study, A1AT-OE increased the levels of PERK and IRE1α signaling-related proteins, whereas inhibition of either PERK or IRE1α largely prevented the A1AT-OE-induced expression of HTRA1. These findings suggest that A1AT may regulate HTRA1 expression through PERK and IRE1α signaling in the extravillous trophoblast.

A1AT is produced by various cell types, including hepatocytes, epithelial cells, and immune cells. Notably, A1AT administration improves the survival rate of mice with peritonitis and sepsis [18]. A1AT expression has been shown to be lower in the placentas of patients with PE than in normal placentas [19,20,21,22,23]. Similarly, A1AT expression is low in a mouse model of PE and high blood pressure, though these symptoms are improved by the administration of A1AT [19]. In the present study, A1AT-OE increased ER stress-mediated invasion and HTRA1 levels, and A1AT-KD reduced invasion. Furthermore, A1AT-OE or A1AT administration increases the migration and invasion of human umbilical vein endothelial cells from women with PE [19]. Conversely, it has been reported that A1AT expression is high in placentas from patients with PE [24] and that the urinary A1AT concentration is high in women with PE [5,25,26]. Our data suggest that A1AT regulates cell motility via ER stress and HTRA1 expression in trophoblast cells, and that dysregulation may be associated with complications of pregnancy, including PE (Table S1). These findings are consistent with the notion that abnormal A1AT expression affects trophoblast invasion and might aggravate PE. An abnormal immune response between mother and fetus and aberrant placentation can both induce HDP, acting as initiation factors during the early stage of pregnancy. Defective trophoblast invasion, and especially insufficient penetration into the endometrium, leads to HDP. Placenta ischemia may result in oxidative stress and inflammation in extravillous trophoblasts, which triggers and accelerates ER stress. The mechanisms of regulation for A1AT expression are still poorly understood. However, A1AT is known to be post-translationally modified to generate various molecular forms with different natures in response to oxidative stress and inflammatory circumstance [27]. Further studies into the polymorphism of the A1AT protein in placental physiology could identify a prognostic marker and therapeutic target of HDP for clinical use.

## 4. Materials and Methods

### 4.1. Cell Culture

The human extravillous trophoblast HTR8/SVneo cell line was cultured in a Roswell Park Memorial Institute 1640 medium (Fujifilm Wako Pure Chemical Corp., Osaka, Japan), supplemented with 10% fetal bovine serum (Nichirei Biosciences, Inc., Tokyo, Japan) and 1% PSN (100 μg/mL penicillin, 100 μg/mL streptomycin, and 200 μg/mL neomycin; Thermo Fisher Scientific, Waltham, MA, USA) at 37 °C in humidified air containing 5% CO_2_ [28].

### 4.2. Transfection of Small Interfering (si)RNA

HTR8/SVneo cells were transfected with non-targeting control, SERPINA1 (A1AT), or HTRA1 siRNAs (each 50 nM; Sigma-Aldrich, Tokyo, Japan) using Lipofectamine RNAiMAX (Thermo Fisher Scientific) according to the manufacturer’s instructions [29].

### 4.3. Transfection of the SERPINA1 Plasmid Construct

The SERPINA1 (A1AT) expression vector pTCP (BC011991) was purchased from TransOMIC Technologies (Huntsville, AL, USA). The pTCP-SERPINA1 plasmid (1 μg) was transfected into HTR8/SVneo cells by electroporation using the Neon transfection system (Thermo Fisher Scientific) according to the manufacturer’s protocol. Cells were pulsed twice with 1400 V for 20 ms. Transfected cells were selected using puromycin (3 μg/mL).

### 4.4. RNA Extraction and Quantitative RT-PCR

RNA was extracted from cultured cells using an RNeasy Mini Kit (Qiagen, Tokyo, Japan) according to the manufacturer’s protocols. Reverse transcription of the mRNA was performed using a ReverTra Ace qPCR RT Kit (Toyobo, Osaka, Japan), and the cDNA produced was subjected to qPCR amplification in a PowerUP SYBR Green Master Mix (Thermo Fisher Scientific). The primers used are listed in Table 1. Calibration curves were used to determine the amplification efficiencies of each target gene and that of the reference gene, glyceraldehyde-3-phosphate dehydrogenase (GAPDH), which were comparable. Sequence Detection System software v2.3 (Thermo Fisher Scientific) was used to determine the mean crossing threshold (Ct) values for each target [30].

### 4.5. Western Blotting

Cultured cells were lysed using a RIPA buffer (Thermo Fisher Scientific). The constituent proteins were separated by SDS-PAGE and then transferred onto polyvinylidene difluoride membranes (Bio-Rad Laboratories, Hercules, CA, USA) using a Trans-Blot Turbo (Bio-Rad). After blocking with Bullet Blocking One (Nacalai Tesque, Inc., Kyoto, Japan), the membranes were incubated with primary antibodies against A1AT (1:2000; Dako, Tokyo, Japan), HTRA1 (1:2000; Genetex, Irvine, CA, USA), CHOP (encoded by the DDIT3 gene) (1:1000; Cell Signaling Technology, Tokyo, Japan), BiP (encoded by the HSPA5 gene) (1:1000; Cell Signaling Technology), PERK (1:1000; Cell Signaling Technology), IRE1α (1:1000; Cell Signaling Technology), ATF6 (1:1000; Proteintech, Tokyo, Japan), calnexin (CANX, 1:1000; Cell Signaling Technology), or GAPDH (1:5000; Fujifilm Wako Pure Chemical Corp.). Immunoreactive bands were detected using enhanced chemiluminescence (Merck Millipore, Burlington, MA, USA) after incubation with horseradish peroxidase-labeled goat anti-rabbit or anti-mouse IgG (1:5000; Vector Laboratories, Burlingame, CA, USA). Signals were detected using a C-DiGit Blot Scanner (LI-COR), and their band density was quantified using Image Studio DiGit software (version 5.2) [31].

### 4.6. Invasion Assay

The migration of HTR8 cells was assessed using a transwell system (Chemotaxicell; Kurabo, Osaka, Japan) equipped with 8 µm pore size polycarbonate filters. Cells were resuspended in their basal media containing 2% FBS and loaded into the upper compartment, which was coated in Matrigel (Corning, Inc., Corning, NY, USA). The transwells were then placed into 24-well culture plates containing basal media supplemented with 2% FBS for 48 h. The cells that invaded beyond the lower surface of the filters were fixed with cold methanol and stained with DAPI. In each experiment, the numbers of cells were counted in five randomly chosen microscopic fields per filter [32].

### 4.7. Cell Viability and Proliferation Assays

HTR8/SVneo cells (5 × 10^3^) were seeded in 96-well culture plates, and cell viability and proliferation were assessed using the colorimetric WST-8 cell viability assay (Cell Counting Kit-8; Dojindo, Kumamoto, Japan) according to the manufacturer’s protocol [30].

### 4.8. Immunohistochemistry

Normal placental tissue was obtained from women in their third trimester (32 weeks of gestation; *n* = 3) who were undergoing surgery. The use of these tissues was approved by the Clinical Research Ethics Committee of Kyushu University and Tokyo University of Pharmacy and Life Sciences (#1512), and informed consent was provided by the participants. Paraffin sections of the late-pregnancy human placentas were immunostained for HTRA1 and HLA-G, as previously described [33]. Briefly, the paraffin sections were rehydrated, boiled for 20 min in a 10 mM citrate buffer (pH 6.0), and then incubated with a rabbit polyclonal anti-HTRA1 antibody (1:100, GTX53558; GeneTex), mouse monoclonal anti-HLA-G antibody (1:100, ab52455; Abcam, Tokyo, Japan), or normal rabbit IgG (1:100, sc-2027; Santa Cruz Biotechnology, Dallas, TX, USA) as a negative control overnight at 4 °C. Subsequently, the sections were incubated with Histofine Simple Stain MAX-PO (Nichirei Biosciences, Inc.) and then with DAB (Fujifilm Wako Pure Chemical Corp.). The sections were counterstained using hematoxylin.

### 4.9. Reagents

The ER stress inducers tunicamycin and thapsigargin were sourced from Fujifilm Wako Pure Chemical Corp. The UPR inhibitors GSK2606414 (GSK, a PERK inhibitor), Kira6 (an IRE1α inhibitor), and AEBSF (an ATF6 inhibitor) were purchased from Selleck Chemicals (Tokyo, Japan).

### 4.10. Statistical Analysis

Data are expressed as means ± SEMs and were compared using Dunnett’s test. A *P*-value of <0.05 was considered to represent statistical significance. Statistical testing was performed using R software (ver.3.6.2; www.r-project.org (accessed on 30 March 2021)).

## Figures and Tables

**Table 1:** Primers for real-time qPCR analyses.

Name (Accession No.)	Sequence (5′---3′)	Product Length (bp)
*GAPDH* NM_002046.7	AGCCACATCGCTCAGACA	66
	GCCCAATACGACCAAATCC	
*SERPINA1* NM_001127704.2	TCAAGGAGCTTGACAGAGACAC	94
	TCGGTGTCCTTGACTTCAAAGG	
*HTRA1* NM_002775.5	AACACCTACGCCAACCTGTG	127
	GCAAACTGTTGGGATCTTCCTG	
*HTRA2* NM_013247.5	TGAAGGTCACAGCTGGAATCTC	147
	TGGGACTCAGGGTCAGCATC	
*HTRA3* NM_053044.5	ACAAGAAGTCGGACATTGCC	139
	ACTGTGTTCTGTAGGGCGAAG	
*HTRA4*	GACTACGTCCAGATTGATGCC	145
NM_153692.4	ACTGCCTAACTCGATCTGAAGG	
*IL6*	CAGGAGCCCAGCTATGAACT	85
NM_000600.5	AGCAGGCAACACCAGGAG	
*IL8*	AAGCATACTCCAAACCTTTCCA	123
NM_000584.4	CCAGACAGAGCTCTCTTCCA	
*HSPA5*	CTGTCCAGGCTGGTGTGCTCT	143
NM_005347.5	CTTGGTAGGCACCACTGTGTTC	
*DDIT3*	AGAACCAGGAAACGGAAACAGA	67
NM_001195053.1	TCTCCTTCATGCGCTGCTTT	
Spliced *XBP1*	CTGAGTCCGAATCAGGTGCAG	59
NM_001079539.2	ATCCATGGGGAGATGTTCTGG	
*ATF4*	GTTCTCCAGCGACAAGGCTA	88
NM_001675.4	ATCCTGCTTGCTGTTGTTGG	
*ATF6*	CAGACAGTACCAACGCTTATGCC	133
NM_007348.4	GCAGAACTCCAGGTGCTTGAAG	
*MMP2*	AGCGAGTGGATGCCGCCTTTAA	138
NM_004530.6	CATTCCAGGCATCTGCGATGAG	
*MMP9*	GCCACTACTGTGCCTTTGAGTC	125
NM_004994.3	CCCTCAGAGAATCGCCAGTACT	
*CARL*	GACCTCTGGCAGGTCAAGTC	71
NM_004343.4	TCAGCGTATGCCTCATCGT	

## Supplementary Materials

The following are available online at https://www.mdpi.com/article/10.3390/ijms22073683/s1, Table S1. Previous main studies closely associated with this study.

## References

[1] Lim K.H., Zhou Y., Janatpour M., McMaster M., Bass K., Chun S.H., Fisher S.J. (1997). Human cytotrophoblast differentiation/invasion is abnormal in pre-eclampsia. Am. J. Pathol..

[2] Brown C.M., Garovic V.D. (2011). Mechanisms and management of hypertension in pregnant women. Curr. Hypertens. Rep..

[3] Hunt J.M., Tuder R. (2012). Alpha 1 anti-trypsin: One protein, many functions. Curr. Mol. Med..

[4] Tamura K., Takashima H., Fumoto K., Kajihara T., Uchino S., Ishihara O., Yoshie M., Kusama K., Tachikawa E. (2015). Possible Role of alpha1-Antitrypsin in Endometriosis-Like Grafts From a Mouse Model of Endometriosis. Reprod. Sci..

[5] Starodubtseva N., Nizyaeva N., Baev O., Bugrova A., Gapaeva M., Muminova K., Kononikhin A., Frankevich V., Nikolaev E., Sukhikh G. (2020). SERPINA1 Peptides in Urine as A Potential Marker of Preeclampsia Severity. Int. J. Mol. Sci..

[6] Jonigk D., Al-Omari M., Maegel L., Müller M., Izykowski N., Hong J., Hong K., Kim S.H., Dorsch M., Mahadeva R. (2013). Anti-inflammatory and immunomodulatory properties of α1-antitrypsin without inhibition of elastase. Proc. Natl. Acad. Sci. USA.

[7] Frochaux V., Hildebrand D., Talke A., Linscheid M.W., Schlüter H. (2014). Alpha-1-antitrypsin: A novel human high temperature requirement protease A1 (HTRA1) substrate in human placental tissue. PLoS ONE.

[8] Singh H., Endo Y., Nie G. (2011). Decidual HtrA3 negatively regulates trophoblast invasion during human placentation. Hum. Reprod..

[9] Wang Y., Lim R., Nie G. (2019). HtrA4 may play a major role in inhibiting endothelial repair in pregnancy complication preeclampsia. Sci. Rep..

[10] Chambers J.E., Dickens J.A., Marciniak S.J. (2018). Measuring the effects of alpha1 -antitrypsin polymerisation on the structure and biophysical properties of the endoplasmic reticulum. Biol. Cell.

[11] Cubillos-Ruiz J.R., Bettigole S.E., Glimcher L.H. (2017). Tumorigenic and Immunosuppressive Effects of Endoplasmic Reticulum Stress in Cancer. Cell.

[12] Fung T.S., Huang M., Liu D.X. (2014). Coronavirus-induced ER stress response and its involvement in regulation of coronavirus-host interactions. Virus Res..

[13] Ando Y., Kuroda A., Kusama K., Matsutani T., Matsuda A., Tamura K. (2021). Impact of serine protease inhibitor alpha1-antitrypsin on expression of endoplasmic reticulum stress-induced proinflammatory factors in adipocytes. Biochem. Biophys. Rep..

[14] Lee C.L., Veerbeek J.H.W., Rana T.K., van Rijn B.B., Burton G.J., Yung H.W. (2019). Role of Endoplasmic Reticulum Stress in Proinflammatory Cytokine-Mediated Inhibition of Trophoblast Invasion in Placenta-Related Complications of Pregnancy. Am. J. Pathol..

[15] Chen Y.Y., Chuang P.Y., Chen C.P., Chiu Y.H., Lo H.F., Cheong M.L., Huang J.Y., Kuo P.L., Chen H. (2014). Functional antagonism between high temperature requirement protein A (HtrA) family members regulates trophoblast invasion. J. Biol. Chem..

[16] Skorko-Glonek J., Zurawa-Janicka D., Koper T., Jarzab M., Figaj D., Glaza P., Lipinska B. (2013). HtrA protease family as therapeutic targets. Curr. Pharm. Des..

[17] Bertolotti A., Zhang Y., Hendershot L.M., Harding H.P., Ron D. (2000). Dynamic interaction of BiP and ER stress transducers in the unfolded-protein response. Nat. Cell Biol..

[18] Kaner Z., Ochayon D.E., Shahaf G., Baranovski B.M., Bahar N., Mizrahi M., Lewis E.C. (2015). Acute phase protein α1-antitrypsin reduces the bacterial burden in mice by selective modulation of innate cell responses. J. Infect. Dis..

[19] Feng Y., Wang N., Xu J., Zou J., Liang X., Liu H., Chen Y. (2017). Alpha-1-antitrypsin functions as a protective factor in preeclampsia through activating Smad2 and inhibitor of DNA binding 4. Oncotarget.

[20] Feng Y., Xu J., Zhou Q., Wang R., Liu N., Wu Y., Yuan H., Che H. (2016). Alpha-1 Antitrypsin Prevents the Development of Preeclampsia Through Suppression of Oxidative Stress. Front. Physiol..

[21] Feng Y.L., Yin Y.X., Ding J., Yuan H., Yang L., Xu J.J., Hu L.Q. (2017). Alpha-1-antitrypsin suppresses oxidative stress in preeclampsia by inhibiting the p38MAPK signaling pathway: An in vivo and in vitro study. PLoS ONE.

[22] Feng Y.L., Zhou C.J., Li X.M., Liang X.Q. (2012). Alpha-1-antitrypsin acts as a preeclampsia-related protein: A proteomic study. Gynecol. Obstet. Investig..

[23] Twina G., Sheiner E., Shahaf G., Yaniv Salem S., Madar T., Baron J., Wiznitzer A., Mazor M., Holcberg G., Lewis E.C. (2012). Lower circulation levels and activity of α-1 antitrypsin in pregnant women with severe preeclampsia. J. Matern. Fetal Neonatal Med..

[24] Buhimschi I.A., Zhao G., Funai E.F., Harris N., Sasson I.E., Bernstein I.M., Saade G.R., Buhimschi C.S. (2008). Proteomic profiling of urine identifies specific fragments of SERPINA1 and albumin as biomarkers of preeclampsia. Am. J. Obstet. Gynecol..

[25] Buhimschi I.A., Nayeri U.A., Zhao G., Shook L.L., Pensalfini A., Funai E.F., Bernstein I.M., Glabe C.G., Buhimschi C.S. (2014). Protein misfolding, congophilia, oligomerization, and defective amyloid processing in preeclampsia. Sci. Transl. Med..

[26] Gerasimova E.M., Fedotov S.A., Kachkin D.V., Vashukova E.S., Glotov A.S., Chernoff Y.O., Rubel A.A. (2019). Protein Misfolding during Pregnancy: New Approaches to Preeclampsia Diagnostics. Int. J. Mol. Sci..

[27] Lechowicz U., Rudzinski S., Jezela-Stanek A., Janciauskiene S., Chorostowska-Wynimko J. (2020). Post-Translational Modifications of Circulating Alpha-1-Antitrypsin Protein. Int. J. Mol. Sci..

[28] Wongwananuruk T., Sato T., Kajihara T., Matsumoto S., Akita M., Tamura K., Brosens J.J., Ishihara O. (2016). Endometrial androgen signaling and decidualization regulate trophoblast expansion and invasion in co-culture: A time-lapse study. Placenta.

[29] Kusama K., Tamura K., Bai H., Sakurai T., Nishi H., Isaka K., Imakawa K., Yoshie M. (2018). Exchange protein directly activated by cAMP (EPAC) promotes transcriptional activation of the decidual prolactin gene via CCAAT/enhancer-binding protein in human endometrial stromal cells. Reprod. Fertil. Dev..

[30] Kusama K., Miyagawa M., Ota K., Kuwabara N., Saeki K., Ohnishi Y., Kumaki Y., Aizawa T., Nakasone T., Okamatsu S. (2020). Cordyceps militaris Fruit Body Extract Decreases Testosterone Catabolism and Testosterone-Stimulated Prostate Hypertrophy. Nutrients.

[31] Kusama K., Bai R., Imakawa K. (2018). Regulation of human trophoblast cell syncytialization by transcription factors STAT5B and NR4A3. J. Cell. Biochem..

[32] Yoshie M., Kashima H., Bessho T., Takeichi M., Isaka K., Tamura K. (2008). Expression of stathmin, a microtubule regulatory protein, is associated with the migration and differentiation of cultured early trophoblasts. Hum. Reprod..

[33] Kusama K., Yoshie M., Tamura K., Daikoku T., Takarada T., Tachikawa E. (2014). Possible roles of the cAMP-mediators EPAC and RAP1 in decidualization of rat uterus. Reproduction.

