# Supplementary material for: Alpha-1 Antitrypsin-Induced Endoplasmic Reticulum Stress Promotes Invasion by Extravillous Trophoblasts
Source: Int J Mol Sci. 2021 Apr 1;22(7):3683. doi: 10.3390/ijms22073683 (PMC8037753; doi:10.3390/ijms22073683)
Supplement: Supplementary file 1 [file ijms-22-03683-s001.zip › Supplementary materials.pptx]

## Slide 1
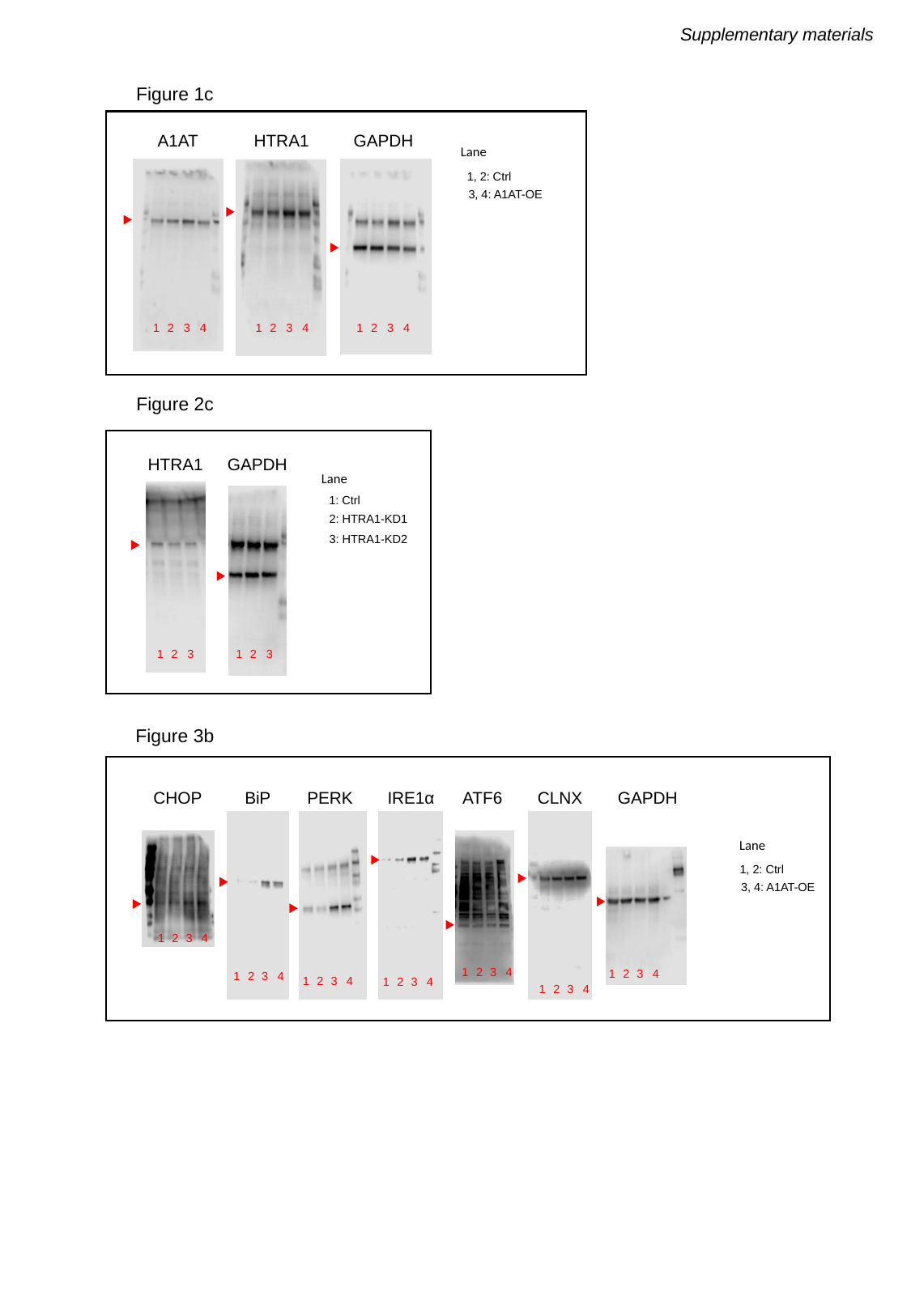

Supplementary materials
Figure 1c
A1AT
HTRA1
GAPDH
Lane
1, 2: Ctrl
3, 4: A1AT-OE
1
2
3
4
1
2
3
4
1
2
3
4
Figure 2c
HTRA1
GAPDH
Lane
1: Ctrl
2: HTRA1-KD1
3: HTRA1-KD2
1
2
3
1
2
3
Figure 3b
CHOP
BiP
PERK
IRE1α
ATF6
CLNX
GAPDH
Lane
1, 2: Ctrl
3, 4: A1AT-OE
1
2
3
4
1
2
3
4
1
2
3
4
1
2
3
4
1
2
3
4
1
2
3
4
1
2
3
4
