# Supplementary material for: Alpha-1 Antitrypsin-Induced Endoplasmic Reticulum Stress Promotes Invasion by Extravillous Trophoblasts
Source: Int J Mol Sci. 2021 Apr 1;22(7):3683. doi: 10.3390/ijms22073683 (PMC8037753; doi:10.3390/ijms22073683)
Supplement: Supplementary file 1 [file ijms-22-03683-s001.zip › Table S1.docx]

Table S1. Previous main studies closely associated with this study

| **Main findings** | **Reference** |
| --- | --- |
| A1AT acts as a preeclampsia- related protein | [22] |
| A1AT prevents development of preeclampsia via suppression of oxidative stress | [19], [20], [21] |
| A1AT expression is low in a mouse model of PE and high blood pressure | [19], [21] |
| PE and high blood pressure are improved by the administration of A1AT | [19] |
| A1AT serves as a biomarker of preeclampsia severity | [5], [24] |
| Lower circulation levels and activity of A1AT in severe preeclampsia | [23] |
| Protein misfolding by A1AT during pregnancy promotes preeclampsia | [25], [26] |
| Possible role of A1AT in endometriosis-like grafts from a mouse model of endometriosis | [4] |
| HTRA1 serves as a substrate for A1AT in human placental tissue | [7] |
| HTRA family members regulate trophoblast invasion | [15] |
